# Single Marker and Haplotype-Based Association Analysis of Semolina and Pasta Colour in Elite Durum Wheat Breeding Lines Using a High-Density Consensus Map

**DOI:** 10.1371/journal.pone.0170941

**Published:** 2017-01-30

**Authors:** Amidou N’Diaye, Jemanesh K. Haile, Aron T. Cory, Fran R. Clarke, John M. Clarke, Ron E. Knox, Curtis J. Pozniak

**Affiliations:** 1 Department of Plant Sciences and Crop Development Centre, University of Saskatchewan, Saskatoon, Saskatchewan, Canada; 2 Semiarid Prairie Agricultural Research Centre, Agriculture and Agri-Food Canada, Swift Current, Saskatchewan, Canada; Mahatma Phule Krishi Vidyapeeth College of Agriculture, INDIA

## Abstract

Association mapping is usually performed by testing the correlation between a single marker and phenotypes. However, because patterns of variation within genomes are inherited as blocks, clustering markers into haplotypes for genome-wide scans could be a worthwhile approach to improve statistical power to detect associations. The availability of high-density molecular data allows the possibility to assess the potential of both approaches to identify marker-trait associations in durum wheat. In the present study, we used single marker- and haplotype-based approaches to identify loci associated with semolina and pasta colour in durum wheat, the main objective being to evaluate the potential benefits of haplotype-based analysis for identifying quantitative trait loci. One hundred sixty-nine durum lines were genotyped using the Illumina 90K Infinium iSelect assay, and 12,234 polymorphic single nucleotide polymorphism (SNP) markers were generated and used to assess the population structure and the linkage disequilibrium (LD) patterns. A total of 8,581 SNPs previously localized to a high-density consensus map were clustered into 406 haplotype blocks based on the average LD distance of 5.3 cM. Combining multiple SNPs into haplotype blocks increased the average polymorphism information content (PIC) from 0.27 per SNP to 0.50 per haplotype. The haplotype-based analysis identified 12 loci associated with grain pigment colour traits, including the five loci identified by the single marker-based analysis. Furthermore, the haplotype-based analysis resulted in an increase of the phenotypic variance explained (50.4% on average) and the allelic effect (33.7% on average) when compared to single marker analysis. The presence of multiple allelic combinations within each haplotype locus offers potential for screening the most favorable haplotype series and may facilitate marker-assisted selection of grain pigment colour in durum wheat. These results suggest a benefit of haplotype-based analysis over single marker analysis to detect loci associated with colour traits in durum wheat.

## Introduction

Marker-assisted selection (MAS) is increasing in use in plant breeding as a means to enrich selections from segregating populations for desirable alleles influencing economically important traits. In durum wheat (*Triticum turgidum* L. var *durum*), most MAS has focused on selection of traits controlled by single genes or large effect quantitative trait loci (QTL) [1]. Identification of robust markers is becoming easier because of the availability of high-density genetic maps (e.g., [2–4]). Although several QTL were reported in the literature, relatively few are practically used in breeding programs [5]. Reasons for their lack of practical use are mostly due to the difficulties with context dependencies caused by genotype-environmental interactions and/or epistasis, to the limitations of sampling bi-parental populations with multi-genic traits, and to lack of follow-through research to validate identified QTL [6–8]. Identification of marker-trait associations using association mapping techniques, could avoid some of these context dependencies.

Association mapping (AM) is a complementary strategy to QTL mapping to identify associations between genotype and phenotype [9] and is based on linkage disequilibrium (LD) in a collection of unrelated individuals. In contrast to bi-parental mapping, AM allows a broader population from which to sample multiple alleles and to map with higher resolution [9, 10]. Most AM studies test correlations between a single marker and phenotypes. However, because patterns of variation within genomes are inherited as linkage blocks [11–13], clustering markers into haplotypes is gaining acceptance in genome-wide association studies.

Advances in high-throughput genotyping technologies have made SNPs markers of choice for genome-wide association studies. SNPs are the most abundant class of sequence variability in the genome and thus have the potential to provide the highest map resolution (Jones et al. 2007). However, SNPs are usually bi-allelic so each provides less polymorphism information content (PIC) than markers such as SSRs (multi- allelic), thus marker density must be increased. This limitation can be overcome by merging SNPs into haplotypes (Lu et al. 2012). Haplotype-based analyses have been successfully carried out mostly in human genetics due to the availability of data from the HapMap project [14, 15]. Similar efforts are gaining ground in various crops such as maize [16–18], rice [19–22] and soybean [23–26]. In wheat, haplotype analyses were performed for QTL or marker-trait association studies [27–29], pattern of genetic variations [30–32] and gene diversity [33–35]. However, only a relatively low number of SNPs and/or SSR markers were used for marker-trait association studies.

Various arguments advocating for haplotype-based analysis rather than single marker analysis have been proposed. In particular, haplotype-based analysis could capture epistatic interactions between SNPs at a locus [36, 37]; provide more information to estimate whether two alleles are identical by descent [38]; elucidate the exact biological role played by neighbouring amino-acids on a protein structure [39]; reduce the number of tests and hence the type I error rate [40]; capture information from evolutionary history [41]; and provide more power than single marker when an allelic series exists at a locus [42–45]. The fundamental question that arises from all these rationales is to know whether the power and accuracy of association mapping can be improved by grouping SNPs into haplotype blocks (see [46] for a review). Intuitively, one could expect haplotypes to be more powerful due to the simultaneous use of multiple markers information [47–49]. Simulation studies have shown that clustering of markers into haplotypes can provide greater QTL detection power and mapping accuracy than single markers [43, 50–52], and this was supported in empirical studies [17, 18, 53–60]. Haplotype-based approach improves prediction accuracy compared with the individual SNP approach [61–63]. In contrast, a few studies found no apparent advantage of haplotype-based analysis over individual SNP analysis [64–66] for detecting QTL. The outcome of the haplotype-based analysis could change under different models relating genotype to phenotype or under different demographic scenarios [67]. Indeed statistical adjustments for population structure and inclusion of kinship relationships is critical to reduce type I error rates of association mapping studies regardless of a haplotype or single marker approach is used [68–70].

There are various criteria for defining haplotype blocks [12, 46, 57, 71, 72]. In particular, haplotype blocks can be defined using a sliding window [28, 57, 73–75] or combining SNPs within a specific window size [22, 58, 76]. Studies in barley provided good support for the use of simple overlapping sliding windows of three SNPs [57]. Other studies proposed different numbers of SNPs for sliding windows, ranging from 2 to 10 SNPs [28, 77–80]. Although this approach is easy to implement, it could potentially lead to large degrees of freedom in the test statistic due to the large number of haplotypes.

A key factor in the success of whole-genome association mapping remains adequate marker coverage across the genome because sparse coverage reduces the power for marker identification [81]. However, the extent of genotyping required increases with rapid LD decay. Linkage disequilibrium is higher in autogamous species due to lower effective recombination [82]. In durum wheat, LD is limited to distances of 2 to 5 cM but is not uniform along chromosomes [83]. Advances in sequencing and genotyping technology allow generation of large amounts of SNP data and the Illumina 90,000 iSelect SNP chip [4] allows development of several robust high-density genetic maps of tetraploid wheat (see [84] for review). We published the first high-density SNP consensus map which anchored over 35,000 SNP markers to all 14 durum wheat chromosomes [3]. The average marker density was 0.079 cM/marker for the B genome and 0.101 cM/marker for the A genome, which provides a framework for association mapping. Because the majority of mapped SNPs are gene-derived markers, this map provides valuable anchor points for post-mapping genetic analysis of the loci and QTL [3].

Improvement of yellow pigment (YP) concentration in durum grain is targeted globally by breeding programs due to increased market demand for bright yellow colour of semolina and pasta products (see [85] for review). The genetics of YP is complex [86], and is due to carotenoid pigment content in the endosperm. Quantitative trait loci were detected on all chromosomes of the durum genome, and genomic regions housing known YP QTL were confirmed on groups 1, 2 and 3 chromosomes [87]. Quantitative trait loci analyses for YP was performed in both hexaploid [88–91] and durum wheat [86, 92–98]. In particular, a major QTL of YP was detected on chromosome 7AL by Parker et al. [90], explaining 60% of the genetic variation and supported by other studies [89, 91, 94, 95, 97, 98]. By contrast, Elouafi et al. [93] detected a major QTL of YP on 7B accounting for 53% of the total variation, and also reported by Kuchel et al. [88], Pozniak et al. [86], Zhang et al. [97] and Zhang and Dubcovsky [98]. Several minor QTL for YP were detected on chromosomes 3A [90], 4A and 5A [99], 2A, 4B and 6B [86], 4B and 6B [97], 1A, 3B and 5B [95], 3B and 5B [94], 1A, 1B, 3B and 4A [91].

Reimer et al. [87] utilized a genetically diverse collection of cultivars and breeding lines collected from global breeding programs, and performed association mapping for grain YP concentration. Although AM was successful at identifying QTL, we have not applied these to MAS because validation experiments showed most QTL did not explain sufficient proportions of phenotypic variation in our locally-adapted breeding materials. In addition, several of the QTL that we discovered were specific to lines from the diverse collection but most were identical by state in our breeding material, despite large phenotypic differences in trait expression [100, 101]. One strategy to overcome such limitations is to perform association mapping in locally-adapted breeding material [5]. Phenotypic data collected during the course of testing of inbred lines within a breeding program, often with replication over environments, is a valuable resource for discovery of marker associations because these lines are expected to carry a high proportion of relevant, desirable alleles. However such phenotypic data sets are usually unbalanced because breeders tend to cull materials throughout the breeding cycle, making exploitation of such data complicated [5]. Utilization of a common set of check cultivars over successive breeding cycles in combination with mixed models which incorporate correlations among environments can be used to estimate best linear unbiased estimates (BLUEs) for individual lines and these could then be used to evaluate marker-trait associations. The utility of this approach has been demonstrated for durum wheat [101, 102], bread wheat [103], barley [104–107], potato [108] and sugarcane [109].

Taken together, the recent advances in SNP marker detection in durum wheat and robust phenotypic data collected from our breeding programs [100, 101] provided the opportunity to further assess association mapping strategies of practical use in a breeding program. Also, the availability of a high-density SNP consensus map allows the opportunity to assess haplotype based approaches for AM in durum wheat. The main objective of this study was to compare the two mapping approaches to explore the potential of haplotype-based analysis in durum wheat and to identify genomic regions associated with pigment colour in semolina and pasta.

## Materials and Methods

### Plant material

One hundred and sixty-nine durum lines were selected for the study (S1 Table) from the official Canadian durum cultivar registration trial grown in Canada between 1999 and 2013. Phenotypic data and trials were described in previous reports [101, 110]. Candidate lines were tested for one to three years but only lines with at least two years of data were included in the present study. Each trial included check cultivars; AC Avonlea [111], AC Morse, AC Navigator [112] and Strongfield [113] since 1999, and Commander [114] added in 2001. The checks AC Morse and Commander were dropped in 2013 and the new check Brigade [115] was brought in. Trials were arranged in lattice designs with four replications, except in 2013 where most locations comprised three replications.

### Quality analyses

End-use quality traits were measured on composite grain samples of locations within years. The composites included locations with acceptable physical condition (commercial grade Canada Western Amber Durum #3 or better), and blended to give a target grain protein concentration of about 13%. Yellow pigment (parts per million) of semolina was measured using the AACC method 14–50 (AACC 2000). Colour of semolina and of pasta dried at 70°C was measured with a Minolta CR–200 Chroma Meter (Minolta, Japan) equipped with a 50 mm measuring head to assess CIELAB a* and b* colour space units. Semolina a* measurement was discontinued after 2008. The colour loss during pasta manufacture was estimated by regressing pasta b* on semolina b* [101]. The residuals for each genotype, actual minus predicted values, were used as a measure of colour loss in the analysis. Positive residuals indicate less pigment loss than the population average, while negative residuals indicate greater than average loss. The data were analysed with SAS version 9.3 [116] Proc Mixed using lines (fixed) with years (random) as replication to generate lsmeans. The analyses included all genotypes tested in the registration trial (approximately 300), not just those genotyped, so as to provide a better estimate of random variances and covariances. Pearson’s correlations were performed among the lsmeans of the traits.

### SNP genotyping and genetic diversity analysis

Genomic DNA was extracted from fresh young leaf tissue using a modified CTAB method [117]. DNA was quantified using PicoGreen (Invitrogen) fluorescence assay, and diluted to 50 ng/μl. Genotyping was performed according to the method published previously [4]. The 90K iSelect assay chips were run on an Illumina HiScan for imaging and the resulting data were loaded into GenomeStudio v2011.1 software (Illumina) for SNP calling. After filtering those SNPs with ambiguous calls, having more than 25% missing values, or having MAF < 0.05, a total of 12,234 polymorphic SNP markers were used for analyses. PowerMarker V3.25 software [118] was used to calculate the summary statistics including allele number, allele frequency and PIC.

### Genotyping with Rht-B1b and Lxp-B1 genes

Because the *Lpx-B1* deletion has been associated with reduced colour loss during processing [119, 120], the registration lines were genotyped with a *Lpx-B1* marker. The registration lines were also genotyped with *Rht-B1b*, an allele known to confer semi-dwarf growth habit in wheat [121] because the relatively few semi-dwarf lines in the panel were selected for very high pigment, presenting the possibility of spurious associations. In order to relate the association signals to *Lpx-B1* and *Rht-B1b*, pairwise LD (*r*^2^) was performed between all 4B association signals and these genes using MIDAS software [122].

### Population structure and linkage disequilibrium analysis

Population structure is one of several important factors that strongly influence LD. The presence of population stratification and an unequal distribution of alleles within groups can result in spurious associations [82]. Population structure was estimated using discriminant analysis of principal components (DAPC) as implemented in the Adegenet R package version 1.4 [123]. To avoid unstable results, the maximum number of principal components (PCs) should be ≤ N/3, N being the number of lines [123]. Therefore, 56 PCs were included in the model.

Single nucleotide polymorphism markers having MAF < 0.05 were filtered out prior to estimating the LD because the estimation of LD using *r*^2^ is dependent on allele frequency and rare alleles can inflate the *r*^*2*^ [124]. The LD was estimated as a correlation coefficient (*r*^*2*^) between all pairwise comparisons of loci both genome-wide and at the chromosome level, using the Genetics R package available at http://cran.r-project.org/. The *r*^2^ distribution of loci belonging to different chromosomes was used to calculate a threshold of *r*^2^ for LD which was taken from the parametric 95^th^ percentile of that distribution [125]. The genetic distance corresponding to that *r*^2^ threshold was determined with nonlinear regression by plotting the genetic distance over which LD decayed, using R code written by F. Marroni that is available at http://fabiomarroni.wordpress.com/.

### Marker imputation and haplotype construction

Prior to haplotype construction, missing calls were imputed using the RF regression procedure [126] as implemented in the R package “randomForest” [127, 128]. The RF procedure has been described in detail for imputing missing genotypes for genomic selection [129] and has been successfully used for genetic diversity analysis [130] and genome-wide association studies [131–133].

For haplotype construction, redundant information known to introduce bias [134] was first filtered out using an in-house Ruby script. When two or more SNPs had the same genotype across all breeding lines along the same chromosome, they were represented by a single genotype. Thus, a total of 8,581 SNPs were used for the analysis. The SNPs were sorted by position along each chromosome based on the durum high-density SNP-based consensus map [3]. Those SNPs spanned all 14 chromosomes of durum wheat with an average density of one marker per 0.3 cM (S2 Table). Then, SNPs within a window size of 5.3 cM (estimate of average LD decay) on the same chromosome were combined to form a haplotype block and assigned to the same locus. Loci for each chromosome were named as combination of the prefix ‘*hap*’, the chromosome and an index that is the incrementing number (1 to N, N being the total number of haplotypes) of the haplotype along the chromosome (e.g., *hap_1A_1* and *hap_1B_2* designate the first haplotype on chromosome 1A and the second haplotype on chromosome 1B, respectively). Only 17 haplotypes appeared to be rare (MAF < 0.05) and were excluded from further analyses.

### Association analysis

Marker-trait associations were carried out using the general linear model (GLM) and the mixed linear model (MLM) as implemented in TASSEL software version 3 [135]. In order to control spurious associations, population structure and/or relatedness between individuals were taken into account in both GLM and MLM procedures. The Q matrix was based on the four groups from the discriminant analysis of principal components and the kinship (K) matrix was calculated using TASSEL. To control for experiment-wise error, nominal *P*-values were adjusted according to Storey-Taylor-Siegmund’s adaptive step-up procedure [136] as implemented in the Mutoss R package [137]. A false discovery rate (FDR) of 5% was used for computation and only SNPs and haplotypes having an adjusted *P*-value less than 0.05 were declared significant. The allelic effect of haplotypes and SNPs was estimated as the difference between the mean value of the lines carrying these haplotypes and SNPs, and the mean value of the entire population for each trait. Thus, only SNPs and haplotypes having relatively strong allelic effect were reported.

## Results

### Analysis of phenotypic data

Large phenotypic variation was observed among the breeding lines for all of the traits (Table 1). In particular, pasta a* and semolina pigment values ranged from 1.66 to 5.79 and 6.0 to 12.05, respectively. Significant differences were observed between subpopulations (Table 1). The correlation among colour traits is presented in Table 2. Pasta a* was significantly (P < 0.001) correlated with all of the traits, and ranged from *r* = 0.40 (pigment loss) to 0.69 (semolina pigment). Semolina a* was correlated with only pasta a*. However, semolina pigment exhibited strong correlation with semolina b*, pasta a* and pasta b*. The highest correlation (*r* = 0.96) was observed between semolina pigment and semolina b*.

**Table 1:** Average values (minimum, maximum) of color traits for the whole population (WP) and least squares means by sub-populations (SP).

Traits	WP	SP1 (n = 46)^1^	SP2 (n = 38)	SP3 (n = 35)	SP4 (n = 50)	LSD_.05_^2^
Pasta a*	3.34 (1.66, 5.79)	3.26^a^	3.95^a^	3.28^b^	2.98^c^	0.28
Semolina a*	-3.02 (-3.24, -2.56)	-3.02^ab^	-2.97^a^	-3.03^b^	-3.04^b^	0.05
Pasta b*	64.85 (58.14–71.81)	64.51^b^	66.15^a^	64.71^b^	64.26^b^	1.08
Semolina b*	34.22 (29.39, 39.43)	34.77^a^	34.58^a^	34.06^ab^	33.57^b^	0.76
Pigment loss	-0.08 (-5.39, 6.21)	-0.92^c^	0.89^a^	-0.07^b^	-0.06^b^	0.73
Semolina pigment	8.82 (6.00, 12.05)	9.26^a^	9.18^a^	8.70^b^	8.22^c^	0.47

^1^ Subpopulation size

^2^ Average least significant difference, P<0.05

Values with the same appended letter are not significantly different according to the least significant difference test at p < 0.05 (for each trait). n: Subpopulation size.

* Should be read as star (e.g., Pasta a* is ‘Pasta a star’)

**Table 2:** Correlation coefficients among colour traits for 169 durum lines.

Traits	Semolina pigment	Pasta a*	Semolina a*	Pigment loss	Pasta b*
Pasta a*	0.69***				
Semolina a*	0.15	0.42***			
Pigment loss	0.13	0.40***	0.02		
Pasta b*	0.72***	0.65***	0.07	0.76***	
Semolina b*	0.96***	0.59***	0.09	0.1	0.72***

***highly significant at *P* < 0.001

* Should be read as star (e.g., Pasta a* is ‘Pasta a star’)

### Population structure and LD decay

**Fig 1:**
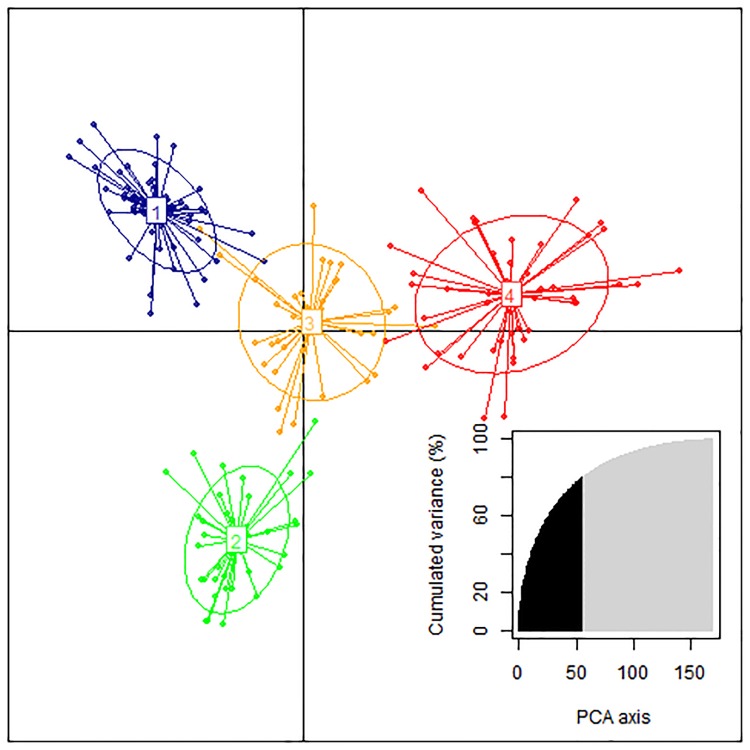
Population structure of the breeding panel as revealed by discriminant analysis of principal components. Each color represents a sub-population. The first 56 axes explained 80% of the total variance.

Four subpopulations among the breeding lines were inferred using discriminant analysis of principal components (Fig 1). The accessions list with their subpopulations is shown in S1 Table. The total amount of genetic variation explained by the first 56 eigenvectors was 80%. Breeding lines were differentiated according to pedigree, source breeding program, and testing year. Subpopulation 1 is largely AC Avonlea [111] and/or Strongfield [113] heritage and comprised on average the most recent lines in the trial. Subpopulation 2 is based on Kyle [138] heritage, with the majority of the lines from the Agriculture and AgriFood Canada, Swift Current program and representing an earlier era of testing than subpopulation 1. Subpopulation 3 contained lines with diverse ancestry from CIMMYT, University of North Dakota, Agriculture and AgriFood Canada, Winnipeg and Swift Current, and University of Saskatchewan. Subpopulation 4 was similar to subpopulation 3 but without the Swift Current component and represented the oldest era of testing of the four groups.

**Fig 2:**
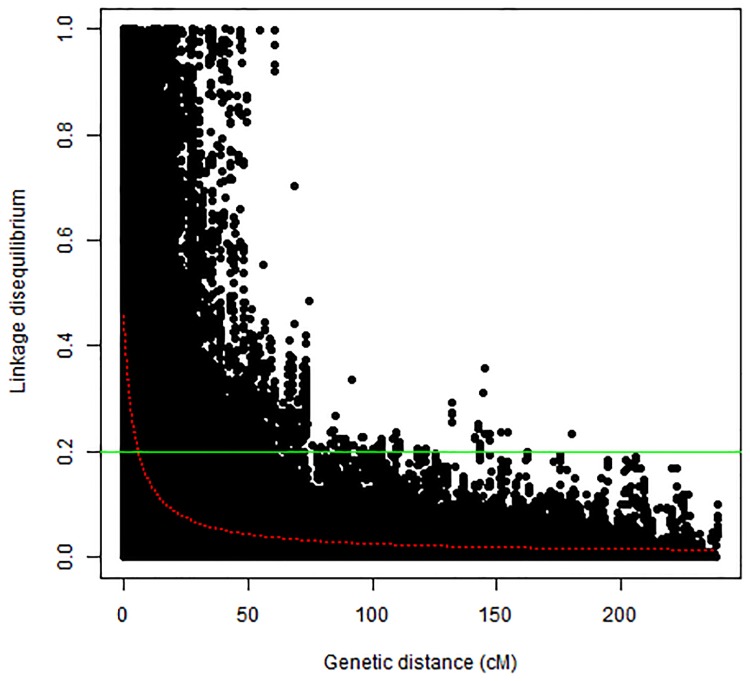
Linkage disequilibrium (LD) scatterplot based on all pairwise comparisons between adjacent loci in the breeding panel. The green horizontal line shows the critical r^2^ while the red curve displays the LD decay.

A total of 12,234 polymorphic SNPs were used to estimate the LD across all chromosomes. The critical *r*^*2*^ value from which the genome-wide LD decayed was estimated at 0.2 (Fig 2). The average genetic distance at which LD across all chromosomes decayed (*r*^*2*^ < 0.2) was 5.3 cM. Nonetheless, that distance varied among chromosomes, from 3.0 (chromosome 4A) to 9.4 cM (chromosome 5B). The LD pattern of all chromosomes is presented in S1 Fig. Only 4% of all pairs of SNPs showed very high LD (*r*^*2*^ > 0.8).

### Allele diversity as revealed by SNPs and haplotypes

**Fig 3:**
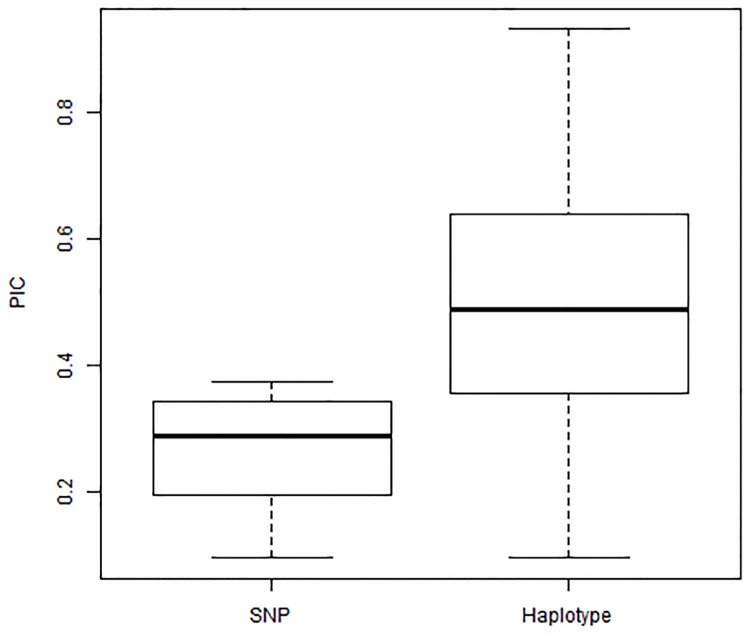
Boxplot of polymorphism information content (PIC) for individual SNP and haplotype. The average PIC was 0.27 for individual SNP and 0.5 for haplotypes.

After imputation, a total of 8,581 SNPs having a minor allele frequency greater than 5% and located on the high-density consensus map were used for analyses. Only 14.2% (1,222/8,581) of the SNPs showed almost equal allele frequencies between their two alternative alleles. The average PIC for these 8,581 SNPs was 0.27, ranging from 0.10 to 0.38 (Fig 3).

A total of 406 haplotype blocks containing 2 to 60 SNPs were generated. Of these haplotype blocks, 4.9% contained two SNPs, 47.5% contained three to nine SNPs and 47.6% had more than 10 SNPs. Haplotype blocks showed a higher level of allele diversity; the average PIC was 0.50, ranging from 0.10 to 0.93 (Fig 3). The number of allele combinations varied from 2 to 161 among haplotype blocks.

### Loci associated with pigment colour

As shown by the quantile-quantile plots (S2 Fig), the MLM (K) and MLM (Q+K) models were significantly better than the GLM naïve and GLM (Q) models in reducing spurious associations. Only the MLM (Q+K) model was kept for the analyses because in general it performed a little better than the MLM (K) model.

**Fig 4:**
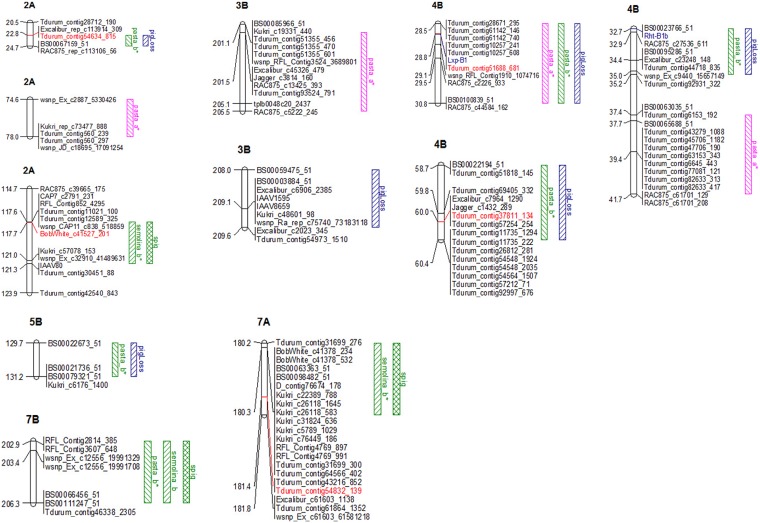
Genomic regions associated with semolina and pasta colour in durum wheat (based on the Maccaferri et al. (2014) map). Markers highlighted in red are those detected by the individual SNP-based analysis.

Single marker-based analysis identified five loci associated with colour components (Table 3, Fig 4). The number of loci varied depending on the trait. Most of the loci revealed by the single marker-based analysis were associated with at least two traits, *Tdurum_contig51688_681* on 4B with pasta a*, pasta b* and pigment loss; *Tdurum_contig54634-815* on 2A with pasta b* and pigment loss; *BobWhite_c41527_201* on 2A and *Tdurum_contig54832_139* on 7A with semolina b* and semolina pigment. Three loci associated with pigment loss were detected on chromosome 2A and 4B, explaining 11.9 to 26.2% of the phenotypic variation. A total of three loci, located on 2A and 4B, were associated with pasta b*, explaining 9.5 to 26.2% of the variation.

**Table 3:** Individual SNP and haplotype loci significantly associated with colour traits.

Trait	Haplotype-based analysis						Single SNP-based analysis					Comparison	
	Haplotype	Position	nbM^1^	P-value	R^2^(%)	Effect	Marker	Position	P-value	R^2^(%)	Effect	IVE^2^(%)	IAE^3^(%)
Pasta a*	*hap_2A_12*	2A (74.6–78)	4	3.69E-04	15.1	1.6							
	*hap_3B_32*	3B (201.5–205.5)	3	1.04E-03	13.4	1.0							
	*hap_4B_6*	4B (28.5–30.8)	9	8.65E-04	35.7	1.6	Tdurum_contig51688_681	4B (28.8)	1.27E-07	19.0	1.6	87.9	0.0
	*hap_4B_8*	4B (37.4–41.7)	9	1.04E-03	27.6	1.7							
Pasta b*	*hap_2A_5*	2A (22.8–24.7)	4	8.08E-04	16.4	3.3	Tdurum_contig54634_815	2A (22.8)	1.37E-04	9.5	3.2	72.6	3.1
	*hap_4B_6*	4B (28.5–30.8)	9	1.62E-04	40.2	5.6	Tdurum_contig51688_681	4B (28.8)	9.66E-10	26.2	5.3	53.4	5.7
	*hap_4B_7*	4B (32.7–35.2)	7	1.51E-03	25.6	4.9							
	*hap_4B_12*	4B (58.7–60.4)	6	6.88E-04	14.1	4.5	Tdurum_contig37811_134	4B (60)	4.48E-05	10.9	3.4	29.4	32.4
	*hap_5B_25*	5B (129.7–131.2)	4	6.80E-04	14.1	3.1							
	*hap_7B_36*	7B (202.9–206.3)	2	2.48E-03	9.3	2.3							
Pigment loss	*hap_2A_5*	2A (22.8–24.7)	4	4.65E-04	17.6	3.5	Tdurum_contig54634_815	2A (22.8)	2.34E-05	11.9	2.4	47.9	79.2
	*hap_3B_33*	3B (208–209.6)	3	5.42E-04	14.6	5.4							
	*hap_4B_6*	4B (28.5–30.8)	9	2.03E-03	33.6	3.7	Tdurum_contig51688_681	4B (28.8)	1.17E-09	26.2	3.7	28.2	27.0
	*hap_4B_7*	4B (32.7–35.2)	7	4.01E-04	28.9	4.3							
	*hap_4B_12*	4B (58.7–60.4)	6	9.15E-05	17.5	3.3	Tdurum_contig37811_134	4B (60)	1.02E-05	13.0	2.6	34.6	26.9
	*hap_5B_25*	5B (129.7–131.2)	4	1.06E-03	13.5	2.1							
Semolina b*	*hap_2A_18*	2A (117.6–121.3)	9	8.63E-03	24.5	2.5	BobWhite_c41527_201	2A (117.7)	8.88E-08	19.2	1.9	27.6	31.6
	*hap_7A_32*	7A (180.2–181.8)	10	5.65E-03	34.6	3.3	Tdurum_contig54832_139	7A (181.4)	5.53E-08	19.8	2.0	74.7	65.0
	*hap_7B_36*	7B (202.9–206.3)	2	3.92E-03	8.5	1.3							
Semolina pigment	*hap_2A_18*	2A (117.6–121.3)	9	4.17E-03	27.5	1.9	BobWhite_c41527_201	2A (117.7)	2.00E-08	21.4	1.4	28.5	35.7
	*hap_7A_32*	7A (180.2–181.8)	10	4.42E-03	35.6	2.3	Tdurum_contig54832_139	7A (181.4)	2.67E-08	21.0	1.4	69.5	64.3
	*hap_7B_36*	7B (202.9–206.3)	2	2.94E-03	8.9	1.0							

^1^nbM: Number of markers in the haplotype

^2^IVE: Increase in variance explained obtained from haplotype-based analysis compared to single SNP-based analysis

^3^IAE: Increase in allelic effect obtained from haplotype-based analysis compared to single SNP-based analysis

* Should be read as star (e.g., Pasta a* is ‘Pasta a star’)

Haplotype-based analysis identified a total of 12 loci associated with pigment colour components (Table 3, Fig 4). Detailed information (number and list of SNPs) on these haplotype loci are presented in S3 Table. Most (8/12) of the loci were associated with at least two colour components. In particular, *hap_4B_6*, *hap_4B_7*, *hap_4B_12* and *hap_5B_25* were associated with pasta b* and pigment loss while *hap_2A_18* and *hap_7A_32* were associated with semolina b* and semolina pigment. For pasta a*, a total of four loci were detected, located on chromosomes 2A, 3B and 4B. For pigment loss, six loci were detected on chromosome 2A, 3B, 4B and 5B. Six loci were detected for pasta b*, located on 2A, 4B, 5B and 7B. Three haplotypes (*hap_2A_18*, *hap_7A_32* and *hap_7B_36*) were associated with both semolina b* and semolina pigment. Over all pigment traits, the percentage of variance explained ranged from 8.5 to 40.2%.

Of the three loci on 4B associated with pigment loss, *Tdurum_contig51688_681* (*hap_4B_6*) showed strong LD (*r*^*2*^ = 0.86) with the lipoxygenase gene *Lxp-B1*, while *BS00023766_51* (*hap_4B_7*) was strongly associated (*r*^*2*^ = 0.92) with the dwarfing gene *Rht-B1b* (Fig 4). These two loci appeared to be independent (*r*^*2*^ = 0.31).

### Comparison of loci identified by single marker- and haplotype-based analysis

The haplotype-based analysis identified a total of 12 loci associated with grain pigment colour traits, including all of the five loci identified by the single marker-based analysis. In particular, the haplotype-based analysis detected at least one additional locus for each trait. The loci not detected by the single marker approach explained in general a relatively small amount of the phenotypic variation.

Haplotype-based analysis improved the amount of the phenotypic variance explained and the allelic effect (Table 3). Overall, there was substantial increase in the phenotypic variance explained (50.4% on average) and allelic effect (33.7% on average). For instance, the locus *hap_4B_6* showed an increase of 87.9% for the phenotypic variation of pasta a*; and the allelic effect of the locus *hap_7A_32* was 64.3% greater than that of the associated SNP for semolina pigment. The associated haplotype loci consisted of 2 to 10 SNPs although the number of SNPs ranged from 2 to 60 among the 406 haplotype blocks.

## Discussion

### Population structure and LD decay

In this study, the discriminant analysis of principal components [139] clustered the 169 breeding lines into four subpopulations. This population structure is in agreement with known differences in pedigree, breeding program source and era of testing in the trials.

The discriminant analysis of principal components successfully unraveled the population structure in germplasm such as cultivated sweet potato [140], rice [141], acacia [142] and sweet cherry [143]. The presence of genetic structure within a population can lead to spurious association signals [134, 144–148]. Understanding the actual population structure of the durum breeding panel was intended to limit the false discovery rate in the association analysis.

The average genetic distance results suggest that the LD mapping using our breeding panel can achieve a resolution of < 5 cM. Few (4%) markers showed very high LD (r^2^ > 0.8). Our results are congruent with those reported in bread wheat [149] and a geographically diverse durum wheat panel where the LD decayed within 5 cM on average [83]. However, a relatively higher (10 cM) LD decay distance was reported in a durum elite collection [150].

### Association mapping based on single marker and haplotypes

We used the 3-SNP sliding windows method and came up with a total of 8,537 haplotype blocks (data not shown) that is markedly greater than the 406 LD-based haplotype blocks we generated and used for analyses. A large number of haplotypes increases the degree of freedom for a test statistic [151]. Intuitively, the type I error rate would be higher for haplotypes derived from the 3-SNP sliding windows compared to the LD-based haplotypes. In addition, the sliding windows approach raises the question of the optimum number of markers to be included in the haplotype. A large window may include too many non-informative markers while a small window may ignore informative markers, both of which will lead to a reduction in testing power [152]. Alternatively, variable-sized sliding windows approaches have been proposed [73, 153–157]. However, most of the variable-sized methods require some computationally intensive phasing program to account for uncertain haplotype phases [158].

Because the optimal window size is always influenced by the underlying LD pattern [154, 159], we constructed haplotypes based on the average LD extent in our material. It is well known that LD patterns are variable across a large genomic region or the whole genome; therefore we also built haplotypes using chromosome-based LD. However, we found no substantial difference in size or number of haplotypes, using the chromosome-based LD distance rather than the average distance of LD decay (5.3 cM), suggesting that taking the average distance is reasonable for analysis. Similarly, the average LD distance has been used to build haplotypes in many studies when LD extent varied among chromosomes (e.g.[58, 75]). An advantage of using the LD-based method is that it avoids taking an arbitrary or suggestive number of markers to be included in the haplotype. This method is relatively easy to implement although it requires a pre-computation of the LD extent in the material under investigation. Haplotype blocks defined according to the LD usually reflect the variation patterns of the genome better than haplotype blocks artificially outlined by a fixed number of SNP [61].

The haplotype-based analysis was superior to the individual SNP analysis because it identified seven more loci associated with colour components. The same loci (*hap_2A_18*, *hap_7A_32* and *hap_7B_36*) detected for semolina pigment and semolina b* were not surprising because these traits showed the highest correlation (*r* = 0.96) amongst traits. Furthermore, the haplotype-based analysis resulted in a substantial increase (68.3% on average) in the phenotypic variance explained. The improvement ranged from an 87.9% increase of phenotypic variance explained for pasta a* by haplotype *hap_4B_6* to 27.8% for pasta b* by *hap_2A_18* compared to the associated single markers. Increases in the amount of phenotypic variance explained attributed to haplotype-based analysis were also reported in other crop species such as barley [57] and maize [75]. Similarly, haplotypes explained up to 80% more of the phenotypic variance for genes in cattle [53]. The increased allelic effect (e.g., 64.3% increased for semolina pigment attributed to *hap_7A_32*) from combining SNPs into haplotypes demonstrated an increase in power over the single marker method. However, no single allelic combination within any haplotype locus was able to select all of the lines having the desirable phenotype. Moreover, in general each haplotype carried more than one favorable allelic series. For example for pasta a*, in addition to the most favorable allelic series (effect = 1.66) of *hap_4B_6*, two other allelic combinations showed good allelic effect on the trait, 1.41 and 1.37. Combinations of several allelic series within each haplotype, as well as the aggregation of the best haplotypes improved ability to select lines having the desirable phenotypes. These results confirm the complex genetic architecture of colour trait in durum.

Haplotype-based analysis was reported to increase the power of detecting QTL compared to single-marker analysis, based on simulated data [43]. Including more marker alleles in haplotypes leads to a higher proportion of the QTL variance being explained [52, 160] and provides additional power to the analysis [45, 161]. However, the haplotype loci detected in this study were not those having the highest number of SNPs. Thus, the power of haplotypes in increasing the variance explained could not be attributed mainly to the number of markers. The informativeness of markers within the haplotypes is more likely to be of greater importance. As functional nucleotide polymorphism (sequence variations responsible for alterations in gene function) databases are becoming available, including the most informative markers in haplotypes could enhance the potential utility of haplotype-based studies [21, 162]. In contrast, Zhao et al. [74] found no apparent advantage of haplotype-based analysis over individual SNP analysis in their simulation study that was designed to resemble the demography and population history of livestock. Lorenz et al. [163] reached similar conclusion but they noted that their conclusion may not be valid under different models relating genotype to phenotype or under different demographic scenarios. Despite of these contradictory results, haplotype-based analysis could play a critical role in association mapping studies in crop plants as recently discussed by Gupta et al. [164].

### Comparison with QTL for pigment from previous reports

In durum wheat, many QTL for yellow pigment content have been reported on different chromosomes [86, 89, 92, 96], of which 4B. The locus *hap_4B_6* on 4B explained 33.6% and 40.2% of the variation of pigment loss and pasta b*, respectively. The locus on 5B (*hap_5B_25*) explained 14.1% of the variance of pasta b*, congruent with the results of Roncallo et al. [96] who reported a QTL associated to flour yellowness on 5B, explaining 12.2% of the phenotypic variance. Other studies reported QTL associated to yellow pigment on 4B in durum [86] and hexaploid wheat [89]. The locus *hap_7A_32* detected on 7A in our study explained only 35.6% of the phenotypic variance of semolina pigment. Similarly, a major QTL for yellow pigment concentration has been reported on 7A in both bread wheat [89, 90, 94] and durum wheat [95, 97, 98], and shown to be associated with the phytoene synthase *Psy-A1* locus. Other studies reported a major QTL for flour yellowness on chromosome 7B [88, 89, 98], supporting the existence of a second gene affecting yellow pigment concentration in the distal region of chromosome arm 7B. However, the locus *hap_7B_36* detected on 7B explained only 8.9% of the variation of semolina pigment in our material.

Our observations of semolina colour and marked by *hap_7A_32* on chromosome 7A and *hap_7B_36* on 7B for semolina b* were similar to those of Roncallo et al. [96] whom recently reported QTL for flour yellow colour on 7A and 7B. The evidence is strong for involvement of these two chromosomes in controlling endosperm pigment with numerous reports of major QTL for yellow pigment on 7A [89, 90, 94, 95, 97, 98] and 7B [88, 89, 98].

The *Lxp-B1* gene has been mapped on chromosome 4B [86, 99, 165] as well as the *Rht-B1b* conferring semidwarfism in durum [166]. Therefore, we evaluated how these loci relate to *Lpx-B1*.*1* and *Rht-B1b* genes. Two of the three loci we identified on 4B associated with pigment loss, and explaining 28.9 to 33.6% of the phenotypic variation, were associated with *Rht-B1b* and *Lpx-B1*. The locus *hap_4B_6* showed strong LD (*r*^*2*^ = 0.86) with *Lxp-B1*.*1* gene with the locus *hap_4B_7* was strongly associated (*r*^*2*^ = 0.92) with the semidwarf height locus *Rht-B1b*. Both *Lxp-B1*.*1* and *Rht-B1b* are known to reside on chromosome 4B [101, 167]. Because *Lxp-B1* and *Rht-B1b* are both on 4BS, there could be undesirable linkage. However, these two loci showed relatively weak (*r*^*2*^ = 0.31) LD, suggesting an independent segregation in our material. Pozniak et al. [101] reached a similar conclusion based on DArT marker assessment of this breeding panel.

Carotenoid degradation (pigment loss) during pasta processing is controlled by lipoxygenases, polyphenol oxidases and peroxidases. The wheat genes isoforms *Lpx-1* and *Lpx-3* are located on chromosome 4, whereas the *Lpx-2* gene is located on chromosome 5 [97, 119, 120, 168–171]. In developing durum kernels, different transcript levels have been reported, with *Lpx-1* transcripts being the most abundant in mature grain [169]. This suggests that the *Lpx-1* gene might have a major role in oxidation of carotenoid pigments during pasta processing. In support to this hypothesis, a major QTL for total lipoxygenase activity, with three copies of the *Lpx-1* gene (*Lpx-B1*.*1*, *Lpx-B1*.*2* and *Lpx-B1*.*3)* has been mapped on chromosome 4BS [97, 99, 120, 168, 172]. Selection for and fixing this allele in all breeding lines could contribute to significantly reduced pigment loss during pasta processing and, consequently, to improve the aesthetic and nutritional qualities of the pasta products.

For pasta a*, the four loci detected on chromosomes 2A, 3B and 4B suggest complex genetic control of pasta redness in durum wheat. To our knowledge, this is the first study of association mapping for pasta a*. Half of the total number of loci associated with pasta a* were located on chromosome 4B. In particular, locus *hap_4B_6* explained 35.7% of the phenotypic variance. This locus also showed strong association with pasta b* and pigment loss. Pasta a* (redness) and pasta b* (yellowness) being correlated (*r* = 0.65), much effort should be put on breaking the LD between them to facilitate selecting against red colour pasta.

## Conclusion

Our results clearly showed that genome-wide association studies could benefit from haplotype-based analysis. The haplotype approach substantially increased the polymorphism information content and detected more loci associated with semolina and pasta pigment. The amount of phenotypic variance explained and the allelic effect were also improved over single marker analysis. In particular, the locus *hap_4B_6* on chromosome 4B was associated with pasta a*, pasta b* and pigment loss; and explained up to 40% of the phenotypic variation. This locus could be a good candidate for tagging the *Lpx-B1* gene. On the other hand, combinations of several allelic series within each haplotype locus, as well as the aggregation of the best haplotypes improved ability to select lines having the desirable phenotypes. The use of haplotype-based analysis in comparison with single marker analysis will provide more insight about the potential of combining SNPs into haplotypes in genome-wide association studies.

## Supporting Information

S1 Table Lines pedigree and subpopulations they belong to, based on the discriminant analysis of principal components. (DOCX)

S2 Table Distribution of SNPs on the durum high-density SNP-based consensus map. (DOCX)

S3 Table Description of haplotypes associated with pigments colour traits. (DOCX)

S1 Fig Linkage disequilibrium (LD) scatterplot based on all pairwise comparisons between adjacent loci belonging to the same chromosome. (DOCX)

S2 Fig Quantile-quantile (Q-Q) plots comparing the distribution of observed versus expected *P*-values for association analyses of colour traits under different statistical models: GLM naïve (blue diamond), GLM_Q (red square), MLM_K (green triangle) and MLM_QK (purple cross). The black dash line represents the null hypothesis of no true association.
(DOCX)
