# Correction: Single Marker and Haplotype-Based Association Analysis of Semolina and Pasta Colour in Elite Durum Wheat Breeding Lines Using a High-Density Consensus Map

**DOI:** 10.1371/journal.pone.0187178

**Published:** 2017-10-24

**Authors:** Amidou N’Diaye, Jemanesh K. Haile, Aron T. Cory, Fran R. Clarke, John M. Clarke, Ron E. Knox, Curtis J. Pozniak

There is an error in Table 3. The effect of marker Tdurum_contig51688_681 for pigment loss should be 2.9 instead of 3.7. Please see the corrected Table 3 here.

**Table 3:** Individual SNP and haplotype loci significantly associated with colour traits.

	Haplotype-based analysis						Single SNP-based analysis					Comparison	
Trait	Haplotype	Position	nbM^1^	P-value	R^2^(%)	Effect	Marker	Position	P-value	R^2^(%)	Effect	IVE^2^(%)	IAE^3^(%)
Pasta a*	*hap_2A_12*	2A (74.6–78)	4	3.69E-04	15.1	1.6							
	*hap_3B_32*	3B (201.5–205.5)	3	1.04E-03	13.4	1.0							
	*hap_4B_6*	4B (28.5–30.8)	9	8.65E-04	35.7	1.6	Tdurum_contig51688_681	4B (28.8)	1.27E-07	19.0	1.6	87.9	0.0
	*hap_4B_8*	4B (37.4–41.7)	9	1.04E-03	27.6	1.7							
Pasta b*	*hap_2A_5*	2A (22.8–24.7)	4	8.08E-04	16.4	3.3	Tdurum_contig54634_815	2A (22.8)	1.37E-04	9.5	3.2	72.6	3.1
	*hap_4B_6*	4B (28.5–30.8)	9	1.62E-04	40.2	5.6	Tdurum_contig51688_681	4B (28.8)	9.66E-10	26.2	5.3	53.4	5.7
	*hap_4B_7*	4B (32.7–35.2)	7	1.51E-03	25.6	4.9							
	*hap_4B_12*	4B (58.7–60.4)	6	6.88E-04	14.1	4.5	Tdurum_contig37811_134	4B (60)	4.48E-05	10.9	3.4	29.4	32.4
	*hap_5B_25*	5B (129.7–131.2)	4	6.80E-04	14.1	3.1							
	*hap_7B_36*	7B (202.9–206.3)	2	2.48E-03	9.3	2.3							
Pigment loss	*hap_2A_5*	2A (22.8–24.7)	4	4.65E-04	17.6	3.5	Tdurum_contig54634_815	2A (22.8)	2.34E-05	11.9	2.4	47.9	79.2
	*hap_3B_33*	3B (208–209.6)	3	5.42E-04	14.6	5.4							
	*hap_4B_6*	4B (28.5–30.8)	9	2.03E-03	33.6	3.7	Tdurum_contig51688_681	4B (28.8)	1.17E-09	26.2	2.9	28.2	27.0
	*hap_4B_7*	4B (32.7–35.2)	7	4.01E-04	28.9	4.3							
	*hap_4B_12*	4B (58.7–60.4)	6	9.15E-05	17.5	3.3	Tdurum_contig37811_134	4B (60)	1.02E-05	13.0	2.6	34.6	26.9
	*hap_5B_25*	5B (129.7–131.2)	4	1.06E-03	13.5	2.1							
Semolina b*	*hap_2A_18*	2A (117.6–121.3)	9	8.63E-03	24.5	2.5	BobWhite_c41527_201	2A (117.7)	8.88E-08	19.2	1.9	27.6	31.6
	*hap_7A_32*	7A (180.2–181.8)	10	5.65E-03	34.6	3.3	Tdurum_contig54832_139	7A (181.4)	5.53E-08	19.8	2.0	74.7	65.0
	*hap_7B_36*	7B (202.9–206.3)	2	3.92E-03	8.5	1.3							
Semolina pigment	*hap_2A_18*	2A (117.6–121.3)	9	4.17E-03	27.5	1.9	BobWhite_c41527_201	2A (117.7)	2.00E-08	21.4	1.4	28.5	35.7
	*hap_7A_32*	7A (180.2–181.8)	10	4.42E-03	35.6	2.3	Tdurum_contig54832_139	7A (181.4)	2.67E-08	21.0	1.4	69.5	64.3
	*hap_7B_36*	7B (202.9–206.3)	2	2.94E-03	8.9	1.0							

^1^nbM: Number of markers in the haplotype

^2^IVE: Increase in variance explained obtained from haplotype-based analysis compared to single SNP-based analysis

^3^IAE: Increase in allelic effect obtained from haplotype-based analysis compared to single SNP-based analysis

* Should be read as star (e.g., Pasta a* is ‘Pasta a star’
