# Supplementary material for: Single Marker and Haplotype-Based Association Analysis of Semolina and Pasta Colour in Elite Durum Wheat Breeding Lines Using a High-Density Consensus Map
Source: PLoS One. 2017 Jan 30;12(1):e0170941. doi: 10.1371/journal.pone.0170941 (PMC5279799; doi:10.1371/journal.pone.0170941)
Supplement: S1 Table — (DOCX) [file pone.0170941.s001.docx]

S1 Table. Lines pedigree and subpopulations they belong to based on the discriminant analysis of principal components.

| **Lines** | **Subpopulation** | **Cross** |
| --- | --- | --- |
| ACAVONLEA | 1 | 8267-AD2A/DT612 |
| DT558 | 1 | D46-649/D76-1057 |
| DT560 | 1 | D99031/ 9682-AP1//DT713 |
| DT561 | 1 | D40-2237/STRONGFIELD |
| DT562 | 1 | DT529/ 9468-DQ*3//STRONGFIELD |
| DT565 | 1 | DT707/D84-1194 |
| DT568 | 1 | CDC VERONA/STRONGFIELD |
| DT574 | 1 | STRONGFIELD/DT780 |
| DT575 | 1 | STRONGFIELD/DT780 |
| DT704 | 1 | AC AVONLEA/DT665 |
| DT706 | 1 | AC AVONLEA/DT665 |
| DT707 | 1 | AC AVONLEA/DT665 |
| DT713 | 1 | AC AVONLEA/DT665 |
| DT757 | 1 | AC AVONLEA/ 9479-BK4 |
| DT777 | 1 | DT691/MONGIBELLO//STRONGFIELD |
| DT778 | 1 | DT719/STRONGFIELD |
| DT780 | 1 | KYLE//9560A-138/94B27-BR1C/3/DT494/4/AC NAVIGATOR/5/STRONGFIELD |
| DT781 | 1 | KYLE//9560A-138/94B27-BR1C/3/DT494/4/AC NAVIGATOR/5/STRONGFIELD |
| DT783 | 1 | 9561-AJ3A/A9800A-014//STRONGFIELD |
| DT786 | 1 | DT716/STRONGFIELD |
| DT788 | 1 | DT719/STRONGFIELD |
| DT789 | 1 | DT719/STRONGFIELD |
| DT790 | 1 | DT719/STRONGFIELD |
| DT791 | 1 | DT719/STRONGFIELD |
| DT798 | 1 | DT719/DHTON 1//STRONGFIELD |
| DT803 | 1 | 9661-AF1D/ 9586-CL5A//STRONGFIELD |
| DT804 | 1 | 9667A-AV6/DT704//STRONGFIELD |
| DT805 | 1 | 9667A-AV6/DT704//STRONGFIELD |
| DT806 | 1 | 9667A-AV6/DT704//STRONGFIELD |
| DT809 | 1 | CD98578-F-1Y-040M-040YRC-8M-1Y-0B/STRONGFIELD//STRONGFIELD |
| DT813 | 1 | 9667B-AA4/DT704//STRONGFIELD |
| DT818 | 1 | 9675-AP2/DT732//STRONGFIELD |
| DT822 | 1 | DT739/STRONGFIELD |
| DT823 | 1 | DT744/A0100L-007//STRONGFIELD |
| DT824 | 1 | DT732/STRONGFIELD |
| DT831 | 1 | STRONGFIELD/DT745 |
| DT832 | 1 | DT749/DT735//STRONGFIELD |
| DT833 | 1 | A0039&DB764D18/STRONGFIELD |
| DT834 | 1 | DT757/STRONGFIELD |
| DT839 | 1 | DT749/STRONGFIELD |
| DT841 | 1 | A0039&DB764D18/STRONGFIELD |
| DT844 | 1 | SACHEM/STRONGFIELD//DT757 |
| DT848 | 1 | DT769/DT751 |
| DT850 | 1 | A0038-360H03D/DT794 |
| Enterprise (DT787) | 1 | DT716/STRONGFIELD |
| Strongfield | 1 | AC AVONLEA/DT665 |
| ACNAVIGATOR | 2 | KYLE/WESTBRED 881 |
| Commander | 2 | W9260-BK03/AC NAVIGATOR//AC PATHFINDER |
| DT555 | 2 | D24-1773/DT705 |
| DT557 | 2 | D99254/ 9469-EG2 |
| DT570 | 2 | CDC VERONA/DT732 |
| DT685 | 2 | 8982-BA5/KYLE |
| DT708 | 2 | DT674/DT665 |
| DT709 | 2 | DT674/DT665 |
| DT716 | 2 | DT663/ 9469 |
| DT717 | 2 | DT663/ 9469 |
| DT720 | 2 | DT666/DT665 |
| DT721 | 2 | DT665/DT488 |
| DT724 | 2 | DT666/DT665 |
| DT727 | 2 | W9262-260D3/DT488 |
| DT728 | 2 | W9262-260D3/DT488 |
| DT731 | 2 | DT668/DT665 |
| DT732 | 2 | DT663/DT677//DT665/AC NAVIGATOR |
| DT733 | 2 | DT663/DT677//DT665/AC NAVIGATOR |
| DT741 | 2 | DT667/DT665 |
| DT742 | 2 | DT667/DT665 |
| DT743 | 2 | WAKOOMA/AC NAVIGATOR//DT665 |
| DT750 | 2 | GREEN_34/AC NAVIGATOR//DT665 |
| DT760 | 2 | 920334/DT675// 9469-FU5 |
| DT767 | 2 | 920334/DT675// 9469-FU5 |
| DT795 | 2 | 94D11-K*3B/9468-CL5// 9688A-245D2 |
| DT796 | 2 | 94D11-K*3B/9468-CL5// 9688A-245D2 |
| DT800 | 2 | 9685-AF1A/DT721 |
| DT810 | 2 | DT721/COMMANDER//DT720 |
| DT811 | 2 | A9843-BE3D/DT733 |
| DT812 | 2 | DT721/COMMANDER//DT720 |
| DT814 | 2 | DT721/COMMANDER//DT720 |
| DT815 | 2 | DT721/COMMANDER//DT720 |
| DT816 | 2 | A0200H-082/DT735//DT733 |
| DT817 | 2 | DT714/DT720//DT732 |
| DT837 | 2 | A9821-JL5/DT732//DT726 |
| DT838 | 2 | A9821-JL5/DT732//DT726 |
| KRONOS | 2 | KRONOS |
| Kyle | 2 | 6962-92-8-5/ 6965-494-1 |
| ACPATHFINDER | 3 | WESTBRED 881/DT367 |
| BRIGADE | 3 | DT513/DT696 |
| DT521 | 3 | DT618/DT616 |
| DT696 | 3 | DT618/DT637//KYLE |
| DT698 | 3 | 8982-TL05/DT662 |
| DT701 | 3 | 8982-BA5C/DT618 |
| DT702 | 3 | 8982-TL05/DT662 |
| DT703 | 3 | 8982-TL05/DT662 |
| DT705 | 3 | AC AVONLEA/DT665 |
| DT710 | 3 | DT618/GREEN_27 |
| DT714 | 3 | DT618/GREEN_27 |
| DT719 | 3 | 8869-AJ4B/W9260-BK03 |
| DT726 | 3 | DT677*2/DUREX//DT662*2/GREEN_27 |
| DT735 | 3 | DT696/AC AVONLEA |
| DT736 | 3 | DT675/DT665//DT662 |
| DT756 | 3 | AC AVONLEA/ACUATICO_1//DT696 |
| DT763 | 3 | DT513/DT696 |
| DT768 | 3 | DT921/ 9475-CX4//DT696 |
| DT769 | 3 | DT921/ 9475-CX4//DT696 |
| DT770 | 3 | DT696/D941515 |
| DT771 | 3 | DT513/DT696 |
| DT772 | 3 | DT513/DT696 |
| DT774 | 3 | 9489B-EK3/DT696 |
| DT775 | 3 | AC PATHFINDER/DT696 |
| DT779 | 3 | DT719/G9574-AZ3E |
| DT792 | 3 | DT719/G9574-AZ3E |
| DT799 | 3 | AC PATHFINDER/DT696 |
| DT802 | 3 | DT707/DT696 |
| DT819 | 3 | COMMANDER/G9574-AZ3E//DT745 |
| DT820 | 3 | COMMANDER/G9574-AZ3E//DT745 |
| DT840 | 3 | A9918-LX2B/STRONGFIELD |
| DT845 | 3 | DT769/DT751 |
| DT851 | 3 | DT770/STRONGFIELD//A0038-360H03D |
| Eurostar | 3 | G9575B-AA09C/DT498//DT691 |
| Transcend (DT801) | 3 | DT707/DT696 |
| ACMELITA | 4 | MEDORA/LLOYD |
| ACMORSE | 4 | RL 7196/DT610 |
| BEN | 4 | D-8024/MONROE |
| CDCVERONA | 4 | D95253/D95212 |
| DT486 | 4 | VIC/DT384//DT471 |
| DT488 | 4 | RL7174/SCEPTRE |
| DT513 | 4 | DT625/DT612 |
| DT518 | 4 | DT634/DT627 |
| DT520 | 4 | DT633/DT612 |
| DT522 | 4 | DT636/DT641 |
| DT523 | 4 | D89052/DT644 |
| DT524 | 4 | D90056/D90302 |
| DT526 | 4 | D91546/D91507 |
| DT527 | 4 | ND74112/WASCANA//DT354 |
| DT528 | 4 | MEDORA/VIC |
| DT529 | 4 | D92288/D91430 |
| DT530 | 4 | D91430/D91630 |
| DT534 | 4 | DT486///DT477//KAMILAROI/KYLE |
| DT535 | 4 | DT665/DT656 |
| DT537 | 4 | D94350/D93108 |
| DT541 | 4 | DT658/DT659 |
| DT546 | 4 | DT514/D97300 |
| DT548 | 4 | D97437/D98074 |
| DT550 | 4 | D98115/AC NAVIGATOR//DT711 |
| DT551 | 4 | D98115/AC NAVIGATOR//DT711 |
| DT552 | 4 | 9469-EG2/DT711 |
| DT666 | 4 | 8262-Y2A/ 8264-F5D |
| DT668 | 4 | 8262-Y2A/ 8264-F5D |
| DT683 | 4 | DT379/DT618//DT474 |
| DT684 | 4 | DT379/DT618//DT474 |
| DT687 | 4 | DT627/2*KYLE |
| DT688 | 4 | DT618/DT642//DT637 |
| DT689 | 4 | DT618/DT642//DT637 |
| DT691 | 4 | DT618/DT642//DT637 |
| DT692 | 4 | 8667-D037A/ 8960-ADV//DT639 |
| DT693 | 4 | DT639/DT637//DT639 |
| DT694 | 4 | DT639/DT637//DT639 |
| DT695 | 4 | DT471/2*KYLE |
| DT699 | 4 | DT618/DT642//DT637 |
| DT700 | 4 | 8667-D037A/ 8960-ADV//DT639 |
| DT711 | 4 | WESTBRED 881/W9260-BK03 |
| DT715 | 4 | WESTBRED 881/W9260-BK03 |
| DT718 | 4 | 92CA#93/// 4B1149/SCEPTRE//DT637 |
| DT737 | 4 | AC AVONLEA/DT665//AC MORSE |
| DT749 | 4 | AC AVONLEA/NAPOLEON |
| DT751 | 4 | AC MELITA/DT662//DT677 |
| DT794 | 4 | KYLE//9560A-138/94B27-BR1C/3/DT494/4/AC NAVIGATOR/5/ 9687-CA4 |
| Hercules | 4 | LD308/LD368//STEWART,USA/RL3380 |
| NAPOLEON | 4 | VIC/DT384//DT471 |
| Plenty | 4 | ND74112/WASCANA//DT354 |
