# Supplementary material for: Single Marker and Haplotype-Based Association Analysis of Semolina and Pasta Colour in Elite Durum Wheat Breeding Lines Using a High-Density Consensus Map
Source: PLoS One. 2017 Jan 30;12(1):e0170941. doi: 10.1371/journal.pone.0170941 (PMC5279799; doi:10.1371/journal.pone.0170941)
Supplement: S2 Table — (DOCX) [file pone.0170941.s002.docx]

S2 Table. Distribution of SNPs on the durum high density SNP-based consensus map.

| Chromosome | Number of markers | Length (cM) | Density (cM/marker) |
| --- | --- | --- | --- |
| 1A | 531 | 150.3 | 0.3 |
| 1B | 1007 | 164.1 | 0.2 |
| 2A | 576 | 211.5 | 0.4 |
| 2B | 789 | 189.3 | 0.2 |
| 3A | 527 | 184.2 | 0.3 |
| 3B | 441 | 209.6 | 0.5 |
| 4A | 415 | 175.8 | 0.4 |
| 4B | 496 | 135.8 | 0.3 |
| 5A | 449 | 218.6 | 0.5 |
| 5B | 686 | 206.2 | 0.3 |
| 6A | 637 | 131.2 | 0.2 |
| 6B | 694 | 156 | 0.2 |
| 7A | 614 | 208.6 | 0.3 |
| 7B | 719 | 211.7 | 0.3 |
| Total | 8581 | 2552.9 |  |
