# Supplementary material for: Single Marker and Haplotype-Based Association Analysis of Semolina and Pasta Colour in Elite Durum Wheat Breeding Lines Using a High-Density Consensus Map
Source: PLoS One. 2017 Jan 30;12(1):e0170941. doi: 10.1371/journal.pone.0170941 (PMC5279799; doi:10.1371/journal.pone.0170941)
Supplement: S3 Table — (DOCX) [file pone.0170941.s003.docx]

S3 Table. Description of haplotypes associated with pigments colour traits.

| Haplotype | Number of SNPs | Favorable allele series | Chromosome | SNP (Position in cM) |
| --- | --- | --- | --- | --- |
| *hap_2A_5* | 4 | ABAB | 2A | Excalibur_rep_c113914_309 (22.8) Tdurum_contig54634_815 (22.8) BS00067159_51 (24.7) RAC875_rep_c113106_56 (24.7) |
| *hap_2A_12* | 4 | BBBA |  | wsnp_Ex_c2887_5330426 (74.6) Kukri_rep_c73477_888 (78.0) Tdurum_contig560_239 (78.0) wsnp_JD_c18695_17091254 (78.0) |
| *hap_2A_18* | 9 | BBBABBBAA |  | CAP7_c2791_231 (117.6) Tdurum_contig11021_100 (117.6) Tdurum_contig12589_325 (117.6) wsnp_CAP11_c838_518859 (117.6) BobWhite_c41527_201 (117.7) Kukri_c57078_153 (121.0) wsnp_Ex_c32910_41489631 (121.0) IAAV80 (121.3) Tdurum_contig30451_88 (121.3) |
| *hap_3B_32* | 3 | BBA | 3B | Jagger_c3814_160 (201.5) tplb0048c20_2437 (205.1) RAC875_c5222_245 (205.5) |
| *hap_3B_33* | 3 | BAB |  | BS00059475_51 (208.0) BS00003884_51 (209.1) Tdurum_contig54973_1510 (209.6) |
| *hap_4B_6* | 9 | AABAABBBB | 4B | Tdurum_contig28671_295 (28.5) Tdurum_contig61142_146 (28.5) Tdurum_contig61142_740 (28.5) Tdurum_contig10257_241 (28.8) Tdurum_contig51688_681 (28.8) wsnp_RFL_Contig1910_1074716 (29.1) RAC875_c2226_933 (29.5) BS00100839_51 (30.8) RAC875_c44584_162 (30.8) |
| *hap_4B_7* | 7 | BAABBBA |  | BS00023766_51 (32.7) RAC875_c27536_611 (32.9) BS00095286_51 (34.4) Excalibur_c23248_148 (34.4) Tdurum_contig44718_835 (34.4) wsnp_Ex_c9440_15657149 (35.0) Tdurum_contig92931_322 (35.2) |
| *hap_4B_8* | 9 | ABBBAABAA |  | BS00063035_51 (37.4) Tdurum_contig6153_192 (37.4) Tdurum_contig43279_1088 (39.4) Tdurum_contig45706_1182 (39.4) Tdurum_contig47706_190 (39.4) Tdurum_contig63153_343 (39.4) Tdurum_contig6645_443 (39.4) Tdurum_contig77081_121 (39.4) RAC875_c61701_129 (41.7) |
| *hap_4B_12* | 6 | BBBAAB |  | BS00022194_51 (58.7) Tdurum_contig69405_332 (59.8) Excalibur_c7964_1290 (60.0) Tdurum_contig37811_134 (60.0) Tdurum_contig11735_1294 (60.4) Tdurum_contig26812_281 (60.4) |
| *hap_5B_25* | 4 | ABAA | 5B | BS00022673_51 (129.7) BS00021736_51 (131.2) BS00079321_51 (131.2) Kukri_c6176_1400 (131.2) |
| *hap_7A_32* | 10 | ABAAABBBBA |  | Tdurum_contig31699_276 (180.2) BobWhite_c41378_532 (180.3) D_contig76674_178 (180.3) Kukri_c26118_1645 (180.3) Tdurum_contig64566_402 (180.3) Tdurum_contig43216_852 (181.4) Tdurum_contig54832_139 (181.4) Excalibur_c61603_1138 (181.8) Tdurum_contig61864_1352 (181.8) wsnp_Ex_c61603_61581218 (181.8) |
| *hap_7B_36* | 2 | AB |  | RFL_Contig2814_385 (202.9) BS00066456_51 (206.3) |
