# Supplementary material for: Single Marker and Haplotype-Based Association Analysis of Semolina and Pasta Colour in Elite Durum Wheat Breeding Lines Using a High-Density Consensus Map
Source: PLoS One. 2017 Jan 30;12(1):e0170941. doi: 10.1371/journal.pone.0170941 (PMC5279799; doi:10.1371/journal.pone.0170941)
Supplement: S1 Fig — (DOCX) [file pone.0170941.s004.docx]

| 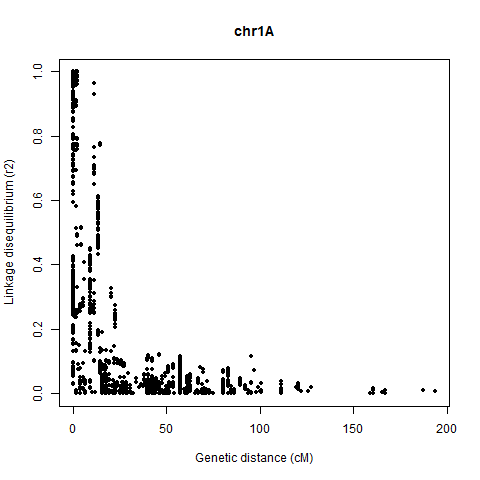 | 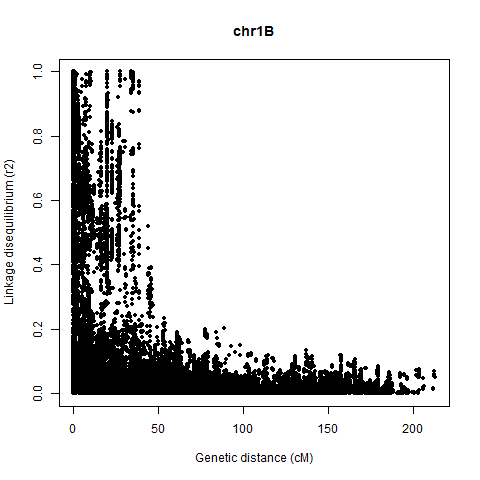 |
| --- | --- |
| 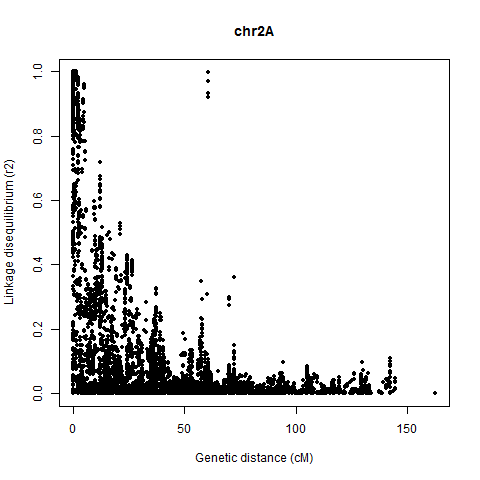 | 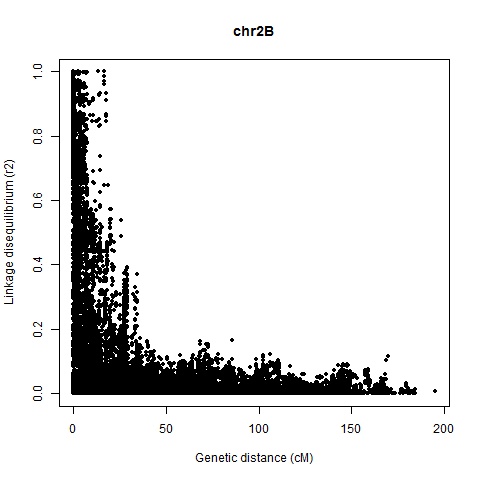 |
| 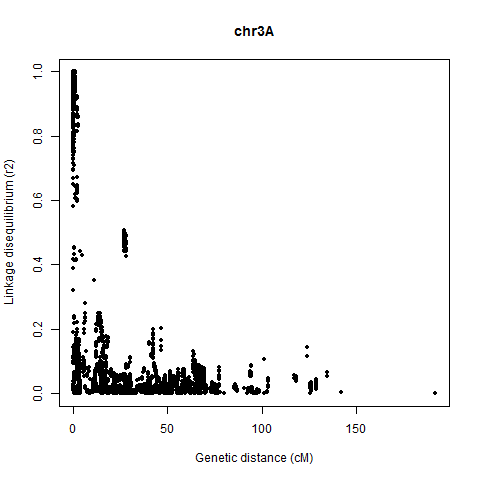 | 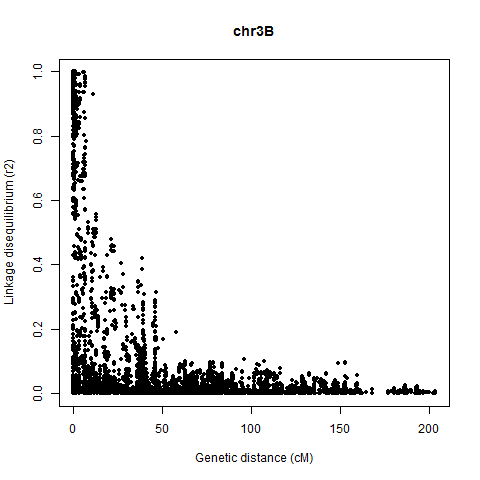 |
| 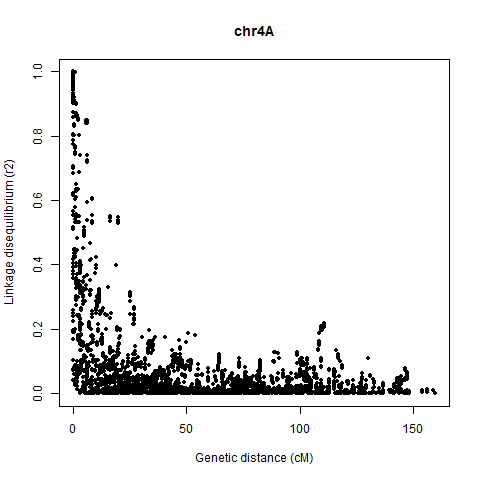 | 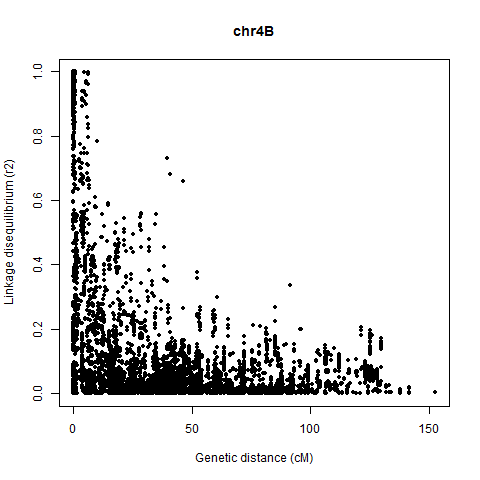 |

| 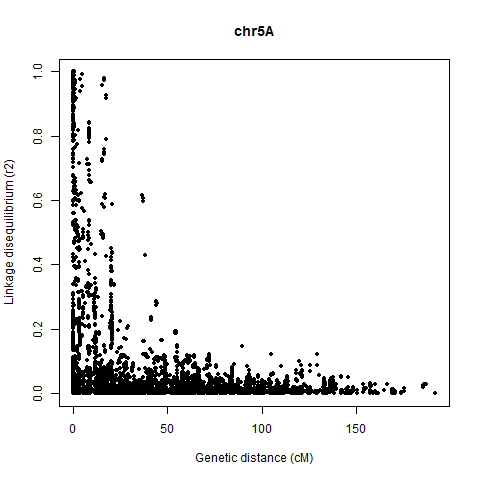 | 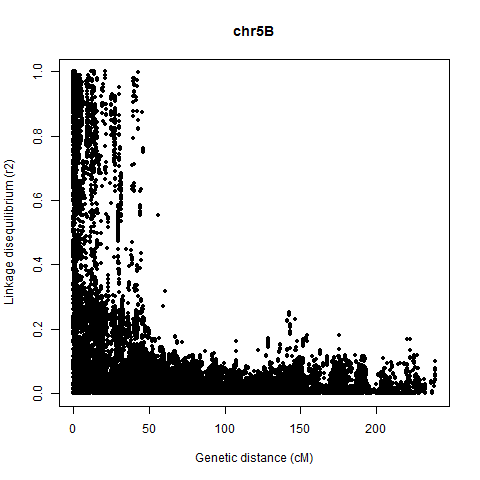 |
| --- | --- |
| 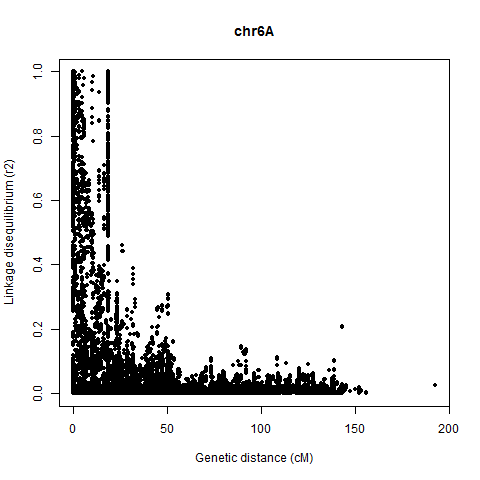 | 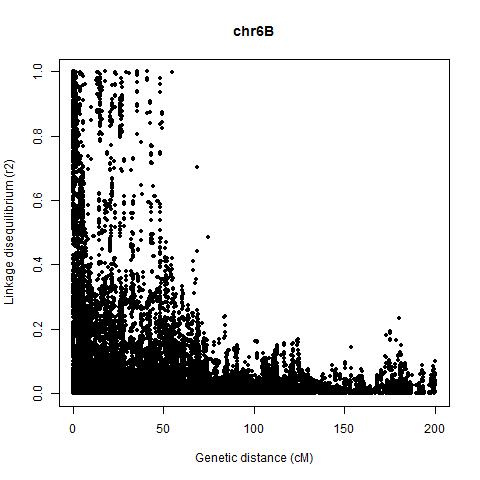 |
| 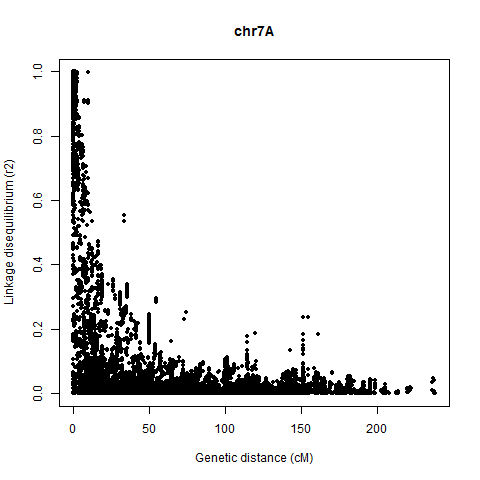 | 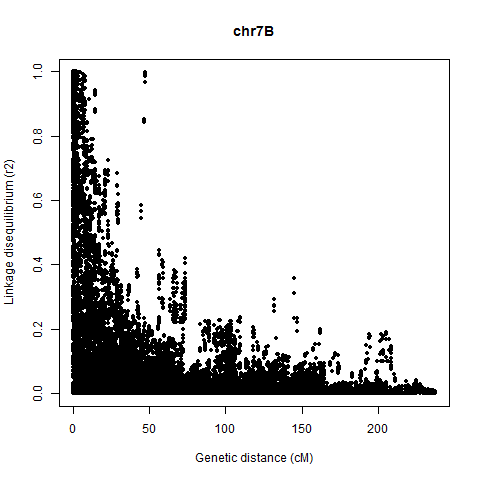 |

**S1 Fig.** Linkage disequilibrium (LD) scatterplot based on all pairwise comparisons between adjacent loci belonging to the same chromosome.
