# Supplementary material for: Single Marker and Haplotype-Based Association Analysis of Semolina and Pasta Colour in Elite Durum Wheat Breeding Lines Using a High-Density Consensus Map
Source: PLoS One. 2017 Jan 30;12(1):e0170941. doi: 10.1371/journal.pone.0170941 (PMC5279799; doi:10.1371/journal.pone.0170941)
Supplement: S2 Fig — The black dash line represents the null hypothesis of no true association. (DOCX) [file pone.0170941.s005.docx]

|  |  |
| --- | --- |
| Pasta a* | Pasta b* |
|  |  |
| Semolina pigment | Pigment loss |
|  |  |
| Semolina b* |  |

**S2 Fig.** Quantile-quantile (Q-Q) plots comparing the distribution of observed versus expected *P*-values for association analyses of colour traits under different statistical models: GLM naïve (blue diamond), GLM_Q (red square), MLM_K (green triangle) and MLM_QK (purple cross). The black dash line represents the null hypothesis of no true association.
